# Supplementary material for: The impact of the digital economy on industrial structure upgrading in resource-based cities: Evidence from China
Source: PLoS One. 2024 Feb 23;19(2):e0298694. doi: 10.1371/journal.pone.0298694 (PMC10889648; doi:10.1371/journal.pone.0298694)
Supplement: Attachment — Submitted filename: report.docx [file pone.0298694.s002.docx]

Reviewer Comments

*The paper presents a research on the influence of the digital economy to the upgrading industrial structure on resource-based cities in China. By using panel data from 2011 to 2020 for 85 resource-based cities in China, the research found that the digital economy is benefit to the upgrading of industrial structure and innovation is the primary mechanism of action. According to the heterogeneity analysis, the results show that there are differences in the impact of the digital economy at the different stages of resource development and the different distance between provincial capital city to resource cities. The paper is well written and the research is well conducted，but there are still some problems that could be improved.*

***Point 1:*** *It is better to add some past literature reviews on the impact of the digital economy on industrial structure upgrading to make the context more coherent.*

***Point 2:*** *It is better to create a variable definition table, so that readers can have a clearer understanding of the explanatory variable, the dependent variable, and the control variable.*

***Point 3:*** *Authors can add more explanation in the distance from the provincial capital city on Section 4.2 to increasing the readability of the article.*

***Point4:*** *Perhaps a placebo test can be performed after completing DID to make the results more stable.*

***Point5:*** *Pay attention to the format of the table, for example, whether the Table 2,3,4,5 needs to indicate the significance of asterisk (*).*

***Point6:*** *There are various types of resource-based cities, and different resources influence the development of the digital economy and the upgrading of industrial structures in distinct ways.*
