# Supplementary material for: The impact of the digital economy on industrial structure upgrading in resource-based cities: Evidence from China
Source: PLoS One. 2024 Feb 23;19(2):e0298694. doi: 10.1371/journal.pone.0298694 (PMC10889648; doi:10.1371/journal.pone.0298694)
Supplement: Attachment — Submitted filename: Response to Reviewers.docx [file pone.0298694.s004.docx]

Reviewer #1: I appreciate for the authors' revision. The existing paper has original content and worthy for publication in the journal. I can recommend it for a possible publication.

# **Authors’ Response:**

The authors gratefully acknowledge the Associate Editor and the Anonymous Reviewers for their detailed and highly constructive criticisms, which greatly helped us to improve the quality and presentation of our manuscript.

Last but not least, we gratefully thank the Reviewer again for his/her outstanding comments and suggestions, which greatly helped us to improve the technical quality and presentation of our manuscript.

Reviewer #2: The quality of this paper has been improved after revision. However, the literature review still lacks logic in its writing. The authors did not combine the relationship between the digital economy and industrial structure upgrading in the literature review, and also lacked a systematic literature review on the transformation of resource-based cities. In addition, the mechanism of the impact of digital economy on the industrial structure upgrading of resource-based cities is still not clear enough, and the authors can make diagrams or construct mathematical models to explain this important issue in depth. Last but not least, the English language is not expressed accurately and some sentences are grammatically incorrect, requiring careful language revision.

# **Authors’ Response:**

The authors gratefully acknowledge the Associate Editor and the Anonymous Reviewers for their detailed and highly constructive criticisms, which greatly helped us to improve the quality and presentation of our manuscript. In the following, we provide detailed, item-by-item, point-by- point responses to all the very interesting issues raised by the Anonymous Reviewers. We have highlighted the main modiﬁcations introduced in the revised manuscript in blue color to help the Associate Editor and the Anonymous Reviewers in ﬁnding the changes made with regards to the previous version. We are indebted to them for their careful assessment and outstanding suggestions for improving our manuscript, which have been extremely helpful in order to enhance its presentation and technical quality.

1. The authors did not combine the relationship between the digital economy and industrial structure upgrading in the literature review, and also lacked a systematic literature review on the transformation of resource-based cities.

# **Authors’ Response:**

Many thanks for your valuable comments. Following your suggestions, We revised the literature review section. We contextualized the transformation of resource-based cities and the relationship between the digital economy and industrial structure upgrading, respectively. The details are as follows:

“

**2 Literature Review**

**2.1 Transformation of resource-based cities**

Many scholars have studied the transformation paths of resource-based cities (Wu et al., 2020; Gu et al., 2022; Sun et al., 2022). Ecological science, innovation, urbanization development, technology, and political incentives all have some influence on the transformation of resource-based cities (Guo et al., 2019; Zhao et al., 2021; Zhang et al., 2018). The government plays a crucial role in the transformational development process of resource-based cities (Liu et al., 2012; Zhang et al., 2018). On the one hand, an appropriate compensation mechanism is essential for resource-based cities (Liu & Zhuang, 2011). Environmental regulation, policy compensation, and financial help are essential for transforming resource-based cities (Lashitew & Werker, 2020; Jiang et al., 2021; Li et al., 2018). In China, government transfer payments promote low-carbon development in resource-based cities (Li & Wang, 2022). On the other hand, laws and regulations can improve the transition performance of resource-based cities by constraining people’s behavior (Li et al., 2013), and the effectiveness of laws and regulations largely depends on the government. In addition, national policies often have a crucial influence on urban transformation (Hu et al., 2016). Several scholars have studied the effects of some policies implemented in China. For example, the civilized city policy has significantly improved energy efficiency in resource-based cities (Li et al., 2022); the resource-based sustainable development policy has contributed to the economic, social, and ecological transformation of resource-based cities (Fan & Zhang, 2021); the new energy demonstration city policy has a significant effect on the green total factor productivity of resource-based cities (Yang et al., 2023).

**2.2 The digital economy and industrial structural upgrading**

Industrial structure upgrading implies the process of evolution from primary to advanced level (Yang et al., 2019). With the acceleration of the global digitalization process, the digital economy has become an essential engine for promoting regional industrial structure upgrading (Pang et al., 2022), and resource-based cities are no exception. Due to the excessive reliance on natural resources, the industrial structure of most resource-based cities is relatively single, which crowds out the space for the development of new industries to a large extent. Moreover, the industrial chain of resource-based cities is not long, and there is an extreme lack of high-value-added products, which also restricts the space for upgrading the original industries. The digital economy has injected a new driving force into the industrial structure upgrading of resource-based cities. On the one hand, digital technology is the core driving force of the digital economy (Wu & Shao, 2022). The application of technologies such as big data and artificial intelligence has realized the intelligence of the production process of traditional industries, promoted the digital transformation of traditional industries (Chang et al., 2023), and driven the upgrading of the value chain (Guan et al., 2021). Second, digital finance improves financial services coverage and broadens enterprises' financing channels in resource-based cities (Ren et al., 2023). The broadened coverage and increased financing channels support industrial structure upgrading. Furthermore, the development of the digital economy has given rise to many new industries (Su et al., 2020), and the development of new industries is a crucial driving force for industrial structure upgrading. Due to long-term resource development, the industrial structure of resources-based cities is dominated by natural resource development and primary processing, with apparent characteristics of industrial privatization. The new industries brought about by the digital economy undoubtedly inject endogenous power for industrial structure upgrading.

”

2. In addition, the mechanism of the impact of digital economy on the industrial structure upgrading of resource-based cities is still not clear enough, and the authors can make diagrams or construct mathematical models to explain this important issue in depth.

# **Authors’ Response:**

Many thanks for your valuable comments. Following the reviewers' comments and referring to related studies, we have created diagrams to illustrate the impact mechanisms clearly. The details are as follows：

“

**3 Theoretical Analysis and hypotheses**

……

……

**3.2 The impact mechanism**

……

……

The digital economy

Industrial structural upgradation

Industry updating

Industry creation

Industry integration

Technological innovation

”

References: Su, J., Su, K., & Wang, S. (2020). Does the Digital Economy Promote Industrial Structural Upgrading?—A Test of Mediating Effects Based on Heterogeneous Technological Innovation. Sustainability, 13(18), 10105. https://doi.org/10.3390/su131810105

3. Last but not least, the English language is not expressed accurately and some sentences are grammatically incorrect, requiring careful language revision.

# **Authors’ Response:**

Many thanks for your valuable comments. In order to improve the accuracy of English expression, we submitted the manuscript to a professional organization to polish the text.

Last but not least, we gratefully thank the Reviewer again for his/her outstanding comments and suggestions, which greatly helped us to improve the technical quality and presentation of our manuscript.
